# Racial disparities in multiple myeloma and access to stem cell transplantation

**DOI:** 10.1038/s41408-024-01097-5

**Published:** 2024-07-22

**Authors:** Joselle Madonna Cook

**Affiliations:** Division of Hematology, Mayo Clinic, Rochester, MN USA

**Keywords:** Health care, Diseases

In this issue of *Blood Cancer Journal*, Ailawadhi et al. explored the sociodemographic variables impacting differences in access and utilization of autologous stem cell transplantation (ASCT) within each of the U.S. census defined racial-ethnic groups [1]. Using the National Cancer Database, records of 111,799 unique patients with multiple myeloma between 2004 and 2013 and who received ASCT were included. Non-Hispanic White (NHW) patients comprised most of the cohort (77.5%), while 15.1% were non-Hispanic Black, 5.2% were Hispanic and 2% were non-Hispanic Asian. Ailawadhi and coauthors identified several consistent themes across the racial-ethnic groups which impacted the utilization of transplant. Over the decade of patients analyzed, there was clear uptrend in the proportions of patients transplanted for all patient groups except for the non-Hispanic Asian cohort. The reasons for the discrepancy among non-Hispanic Asian patients are unclear, but this was notably the smallest sub-population consisting of just 1.8% of the patients analyzed. Generally, patients treated at an academic or research facility, patients who had to traverse greater distance from the treatment facility and those with private payor insurance were more likely to receive ASCT. Higher income increased the odds of ASCT for NHW and Hispanic patients but not for Black or Asian patients. Higher Charlston Comorbidity Index score and older age decreased the odds of receiving autologous stem cell transplantation among all racial ethnic groups.

Inequity in access to healthcare resources is an unassailable reality [2]. Interwoven in the healthcare delivery models are geographic location, patients’ specific socioeconomic status and racial-ethnic disparities, which significantly impact care [2–4]. Multiple myeloma has benefited from a revolutionary wave of novel treatments in the last decade, culminating in improved survival for patients. ASCT, the cornerstone of front-line therapy for eligible patients, is shown to improve progression free survival by 21.3 months in a major randomized clinical trial [5]. Beyond ASCT, new innovative therapies such as chimeric antigen receptor T cell therapy (CART) and bispecific antibodies have emerged to further transform progression free survival. However, just a select subset of patients can routinely access and afford ASCT and the newer cellular approaches. It is *because* of these striking survival gains that the disparities among various patient populations are more glaringly apparent, and the inequities entrenched within our health care systems become exposed. The complicated structure of the US health care system further segregates the care that patients can receive.

Black and Hispanic people are more likely to develop multiple myeloma and present at a younger age than White people [4, 6]. Black patients are about 30% less likely to receive a transplant or receive triplet induction regimens [7, 8]. Black, Asian, and Hispanic patients experience longer time to initiation of treatment than White patients [8–10]. Both Black and Hispanic patients are less likely to receive proactive palliative care [11]. Black patients with myeloma continue to have higher in-hospital mortality related to multiple myeloma compared to both White and Hispanic patients [11]. Black, Asian, and Hispanic patients can have equal, if not superior outcomes if afforded timely assessment and equitable access to care [10].

There are many points to contemplate in this analysis by Ailawadhi and coauthors [1], but these four themes came to the fore as important considerations as we navigate a path towards improving care access for all patients with multiple myeloma.

First, although financial disparities are a factor for access and utilization, there are other important factors at play as well. Higher income brackets did not improve utilization of ASCT among Black and Asian patients. One cannot overlook the role of implicit bias in the referral patterns among physicians [7, 12, 13]. It is often assumed Black patients will not be compliant, will not be able to afford care or will not be interested in clinical trials. These implicit biases are not unique to multiple myeloma but is demonstrated again and again in the high rates of maternal mortality among Black women and the low rates of cardiovascular interventional procedures offered to Black patients [14, 15]. We need to more actively address and eliminate our internal prejudices. Mandatory implicit bias training as part of our continuous medical education is one way to ensure broad exposure to these concepts [16]. Partnerships and engagement in underserved communities could additionally create opportunities for intergroup contact with minoritized populations to further manage biases [16]. As clinicians, it is our responsibility to be cognizant of and exercise cultural sensitivity for each of our patients, to keep their interests (and not our biases) central in medical decision making.

Second, transplantation in myeloma is affected by age in conjunction with other parameters such as comorbidities and performance status. During the period of analysis from 2004 to 2013, for each 10-year increase in age, Ailawadhi et al. found that the odds of receiving ASCT decreased by just over 50%. While older age may be associated with more comorbidities and though older patients may experience more toxicity and morbidity with myeloablative chemotherapy, we now know that age alone should not restrict transplant eligibility. We are starting to better understand the dynamic concepts of fitness and frailty as it pertains to ASCT and chemotherapy dosing. Incorporating frailty in patient assessments and in therapeutic considerations will optimize how we administer chemotherapy and further improve access to not only ASCT but other novel therapies such as CAR-T for our older patients [17].

Third, it is reassuring to see that, regardless of race, patients close to academic, or research centers were more likely to receive ASCT. Patients who lived further away were more likely to receive ASCT in this study [1]. While some studies have shown that greater distance from the treating center significantly limits access to care and correlates with poorer outcomes, the opposite has been demonstrated with respect to access to ASCT both in this study by Ailawadhi and others [1, 18–20]. Ailawadhi et al. did not directly compare distance traveled and insurance status/ income bracket, but it was deduced that patients traveling greater distances for ASCT, probably had the means to travel to a center of expertise [1]. In that regard, the aforementioned patients would have more flexibility for work absences or have the necessary family and social support to make the necessary arrangements. However, not all patients are so privileged. As a medical community we have since recognized the importance of decentralizing such care, particularly as it pertains to clinical trial access. Though there are obvious logistical barriers to expanding transplant access, outreach to community hematologists such that they are aware of transplant facilities and associated resources and/or patient assistance grants is a critical bridge to develop.

Finally, in the United States, access and availability of health care is complex, and related to insurance status; private versus government insurance, managed versus non managed care, health maintenance organization vs preferred provider organization, and the list goes on. This is an important structural barrier to the access to novel therapies and transplantation for the general population. Effecting health care policy change occurs at a federal or state level. It is refreshing to see organizations such as the American Society of Hematology and the American Society of Clinical Oncology implement funded health care policy and congressional fellowships for hematologists and oncologists to get the necessary exposure so that we can effectively lobby for a collective “call to action”.

Of course, there are huge limitations in the application of these racial ethnic groupings, as many do not see themselves adequately represented within these 4 categories of NHW, non-Hispanic Black, non-Hispanic Asian and Hispanic peoples. Furthermore, use of self-identified race overlooks the genetic heterogeneity of people and there has been a shift away from relying on such identifiers [21]. Regardless, the social determinants of healthcare cannot be ignored and are inexorably intertwined in the equitableness of care patients can receive. Defining the variables that impact ASCT utilization from approximately 112,000 patients within each of the major racial ethnic groups by Ailawadhi and coauthors is an important step forward with which we can effect change.
